# Genome Sequences of *Oblitimonas alkaliphila* gen. nov. sp. nov. (Proposed), a Novel Bacterium of the *Pseudomonadaceae* Family

**DOI:** 10.1128/genomeA.01474-15

**Published:** 2015-12-17

**Authors:** Ana C. Lauer, Ainsley C. Nicholson, Ben W. Humrighouse, Brian Emery, Adam Drobish, Phalasy Juieng, Vladimir Loparev, John R. McQuiston

**Affiliations:** a Special Bacteriology Reference Laboratory, Bacterial Special Pathogens Branch, Division of High-Consequence Pathogens and Pathology, Centers for Disease Control and Prevention, Atlanta, Georgia, USA; b Division of Scientific Resources, Centers for Disease Control and Prevention, Atlanta, Georgia, USA

## Abstract

Results obtained through 16S rRNA gene sequencing and phenotypic testing of eight related, but unidentified, isolates located in a historical collection at the Centers for Disease Control and Prevention suggested that these isolates belong to a novel genera of bacteria. The genomes of the bacteria, to be named *Oblitimonas alkaphilia* gen. nov. sp. nov., were sequenced using Illumina technology. Closed genomes were produced for all eight isolates.

## GENOME ANNOUNCEMENT

The Special Bacteriology Reference Laboratory (SBRL) of the Bacterial Special Pathogens Branch at the Centers for Disease Control and Prevention maintains a historical clinical isolate collection of specimens that have been received by the lab since its inception in 1951. Recent efforts to further examine unidentified isolates in the collection revealed eight unidentified isolates, received between 1969 and 1979, that share morphological and biochemical traits. Conventional diagnostics such as 16S rDNA sequencing and analysis via Vitek II systems placed the isolates within the *Pseudomonadaceae* family, however, biochemical data distinguished it from any previously published species. Whole-genome sequencing confirmed that the isolates belong to a novel genus and species, for which the name *Oblitimonas alkaliphila* gen. nov. sp. nov. will be proposed.

The strains (B4199^T^, C6819, C6918, D2241, D3318, E1086, E1148, and E5571) were taken from frozen defibrinated rabbit blood (Hemostat) and grown on heart infusion agar (HIA) with 5% rabbit blood in a candle jar placed in 35°C for 24 h. Isolated colonies were sub-cultured onto HIA with 5% rabbit blood plates. Genomic DNA was isolated via phenol-chloroform-CTAB extraction (1). 2 × 250 paired-end sequencing libraries, with an average insert size of 500 bp, were generated following the manufacturer’s recommendations for the SPRIworks HT fragment library kit (Beckman Coulter, Inc., Brea, CA), labeled with indices from a TrueSeq DNA sample prep kit, and sequenced on an Illumina MiSeq instrument (Illumina, Inc., San Diego, CA).

All sequence reads were analyzed using FastQC v0.10.1. Reads were trimmed based on quality (q = 25) using Trim Galore v0.3.7; any reads of length less than 80 nt were discarded. Using CLC Genomics Workbench v7.5, remaining adaptor sequences and reads of the PhiX174 reference were discarded. *De novo* assemblies (K = 21, 35, 55, and 64) were carried out using the De Bruijn graphing utility in CLC Genomics Workbench. All assemblies were analyzed via QUAST v2.3. The best assembly for each isolate was determined by *N*_50_ length and number of contigs. All assemblies yielded fewer than 30 contigs (Table 1). Each isolate had approximately 300-fold read coverage. Contigs that had low coverage (≤10) were discarded, as were contigs of lengths less than 5000 bp. An optical map was generated for each isolate using the ARGUS whole genome optical mapping system from OpGen (Gaithersburg, Maryland). The optical map assemblies, generated after treating the DNA with BamHI, resulted in single chromosomes that ranged in size from 2,339,193 bp to 2,572,054 bp in length (Table 1). Then, the contigs generated by Illumina sequencing were converted to *in silico* optical maps using the BamHI enzyme, mapped to the isolate-specific OpGen optical map, and ordered in MapSolver. Contigs were ordered based on cut-patterns, and read extension allowed them to be joined manually into single closed-genome contigs (Table 1).

**TABLE 1:** Assembly statistics and accession numbers

	*De novo* assembly				OpGen map		Final assembly				
Isolate	K	No. of contigs	*N*_50_	Length (bp)	No. of chromosomes	Length (bp)	No. of contigs	Length (bp)	No. of genes	Ns^a^	Accession no.
B4199^T^	21	13	261,375	2,458,437	1	2,415,656	1	2,494,031	2,366	0	CP012358
C6819	35	11	307,219	2,265,386	1	2,572,054	1	2,272,143	2,136	0	CP012359
C6918	64	10	218,521	1,900,609	1	2,339,193	1	2,312,033	2,155	0	CP012360
D2441	35	15	211,404	2,407,881	1	2,344,032	1	1,945,418	1,816	976.76	CP012361
D3318	55	10	313,238	2,272,557	1	2,430,457	1	2,294,397	2,142	0	CP012362
E1086	64	17	263,843	2,338,287	1	2,503,601	1	2,391,994	2,219	0	CP012363
E1148	21	9	330,961	2,336,158	1	2,544,555	1	2,377,259	2,222	0	CP012364
E5571	55	22	158,462	2,312,125	1	2,443,236	1	2,397,029	2,273	180.26	CP012365

a Calculated per 100 bp.

### Nucleotide sequence accession numbers.

GenBank accession numbers for each genome are listed in Table 1. The versions described in this paper are the first versions.
